# Self-reported psychological development in cosmetic breast surgery patients

**DOI:** 10.1097/MD.0000000000005620

**Published:** 2016-12-09

**Authors:** María Ángeles Pérez-San-Gregorio, Agustín Martín-Rodríguez, María Jesús Arias-Moreno, María Esther Rincón-Fernández, José Ignacio Ortega-Martínez

**Affiliations:** a Department of Personality, Assessment, and Psychological Treatment, University of Seville, Spain; b Private Practice of Plastic, Reconstructive and Aesthetic Surgery, Seville, Spain.

**Keywords:** anxiety symptomatology, breast augmentation, breast reduction, mastopexy, quality of life

## Abstract

Cosmetic breast surgery is the only therapeutic alternative for psychological and physical complications associated with micromasty, breast ptosis, and macromasty. We analyzed the effects of 2 variables, time, and type of cosmetic breast surgery, on anxiety symptomatology and quality of life.

Following a mixed 3 × 4 design, 3 groups of women with breast augmentation (n = 63), mastopexy (n = 42), and breast reduction (n = 30) were selected and evaluated using the *State-Trait Anxiety Inventory* and the *12-Item Short-Form Health Survey* at 4 different times, the preoperative stage, and at 1, 6, and 12 months postoperative. Pearson's chi square, Welch's U, Games-Howell tests, mixed analysis of variance, and Cohen's *d* and *w* for effect size were calculated.

Results relating to anxiety (state and trait) showed that the time factor was significant (*P* < 0.001) with differences between the preoperative stage (higher anxiety levels) and the 3 postoperative stages: at 1 month (*P* < 0.001), 6 months (*P* < 0.001), and 12 months (*P* < 0.001). In quality of life, type of surgery and time factors were found to have interactive effects on vitality (*P* = 0.044) and role-emotional (*P* = 0.023) dimensions. Compared to the other 2 groups, women who had undergone mastopexy felt worse (vitality) at 1 month since surgery than in the other stages, and better at 6 months since surgery (role-emotional). In the rest of the dimensions, and focusing on the most relevant effect sizes, the type of surgery made a difference in the physical functioning (*P* = 0.005) and role-physical (*P* = 0.020) dimensions, where women who had had breast reduction felt worse than those who had had augmentation. Time also resulted in differences in the physical functioning (*P* < 0.001), role-physical (*P* < 0.001), and bodily pain (*P* < 0.001) dimensions, where women felt worse at 1 month since surgery than during the rest of the stages, as well as in the social functioning dimension (*P* < 0.001) at 1 month, compared to 6 months postoperative.

We conclude that in the long term, women who have cosmetic breast surgery recover their physical and psychological well-being.

## 1 Introduction

Plastic surgery can significantly improve quality of life,^[1]^ where specific problems such as breast hypoplasia (micromasty), breast ptosis or sagging, and breast hypertrophy (macromasty) are associated with significant psychological, and even physical complications, for which plastic surgery (i.e., breast augmentation, mastopexy, and breast reduction) is the only therapeutic alternative.

Several longitudinal studies^[2–6]^ on breast augmentation have shown that its impact on women is positive, increasing their psychosocial and sexual well-being and their satisfaction with their breasts and body image, improving their self-esteem, decreasing depressive symptomatology, and alleviating their eating disorders. In short, women have a better quality of life derived from changes in their sexuality, satisfaction with their body image, and personal wellbeing.^[7]^

Swanson^[8]^ conducted a longitudinal comparative study of the surgical procedure known as mastopexy with groups of participants who underwent mastopexy, augmentation/mastopexy, and breast reduction. During the preoperative evaluation, he found that patients with breast hypertrophy complained of more back, shoulder, and neck pain, and found it harder to perform physical exercise, compared to others. The main reason that these women gave for undergoing surgery was a combination of better cosmetic appearance and less physical discomfort, whereas most mastopexy or augmentation/mastopexy patients prioritized only improvement of the cosmetic component. After surgery, Swanson found that the 3 procedures, with no differences among them, provided patients with a high level of satisfaction (94.3%), a significant increase in physical capacity (96%), improved self-esteem (89.3%), and higher quality of life (69.5%).

Similarly, several longitudinal studies on breast reduction^[9–20]^ have also found the following significant improvements in patients’ quality of life after surgery: increased psychosocial, sexual, and physical well-being; increased satisfaction with the appearance of their breasts and with their body image; improved self-esteem; decreased anxiety-depressive symptomatology; improved breathing; and less pain. These studies demonstrated that after surgery, the quality of life of these patients improved significantly, and that even after breast reduction, the women usually performed more physical exercise and the severity of their eating disorders decreased.^[21]^ Furthermore, breast reduction improves women's body image in such a way that they feel better about themselves and see their bodies as more proportional than before surgery.^[22]^ All of this is reflected in the high degree of satisfaction that most patients report with this surgical procedure.^[23–25]^

In spite of the biopsychosocial benefits associated with cosmetic breast surgery, there are very few psychological studies analyzing the evolution of patients’ well-being by type of surgery. Therefore, in this study, we analyzed anxiety symptomatology and quality of life over time (preoperative and at 1, 6, and 12 months postoperative) by type of cosmetic breast surgery (i.e., breast augmentation, mastopexy, and breast reduction).

## 2 Methods

### 2.1 Patients

A total of 135 women who had undergone cosmetic breast surgery by a single specialist in plastic, cosmetic, and reconstructive surgery from November 2008 to October 2011 participated in the study. Group 1 was made up of 63 women who had undergone breast augmentation, Group 2 of 42 women who had had a mastopexy (breast volume was not modified in 7 and 35 were treated with mastopexy plus a volume increase), and Group 3 was made up of 30 patients who had had breast reduction. The sociodemographic characteristics of the 3 groups are shown in Table 1.

**Table 1:**
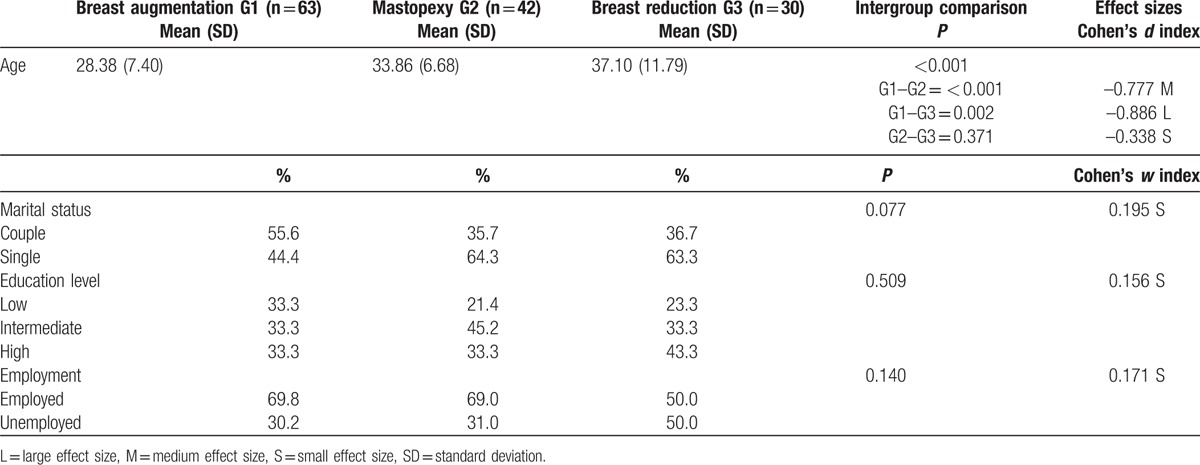
Sociodemographic characteristics of the 3 groups: comparative analyses.

### 2.2 Measurements

The *State-Trait Anxiety Inventory (STAI)*^[26]^ consists of two 20-item scales measuring state and trait-anxiety. All items include a 4-choice response scale (not at all, somewhat, moderately so, very much so) for state-anxiety or (almost never, sometimes, often, almost always) for trait-anxiety. The range of scores for each scale varies from 0 (absence of anxiety) to 60 (maximum anxiety). In this sample, Cronbach's alphas for each stage (preoperative and at 1, 6, and 12 months postoperative) were 0.92, 0.90, 0.92, and 0.91 for the state anxiety subscale and 0.91, 0.89, 0.93, and 0.91 for trait anxiety.

The *12-Item Short-Form Health Survey (SF-12 v.2)*^[27]^ consists of 12 items with either 3- or 5-point Likert-type response scales. It evaluates the following 8 dimensions of health-related quality of life: physical functioning, role-physical, bodily pain, general health, vitality, social functioning, role-emotional, and mental health. The score on each dimensions varies from 0 (worst state of health) to 100 (best state of health). The reliability of the 8 scales varies from 0.73 to 0.87.

### 2.3 Procedure

Patients were selected when they came in to request information on surgery and were assigned to 1 of the 3 groups by type of operation, namely, breast augmentation, mastopexy, or breast reduction.

A sample of 135 women was selected. Inclusion criteria were that they had to be of adult age; never been previously operated on for cosmetic breast surgery, mastectomy, or breast reconstruction after breast cancer; able to read and write sufficiently well to be able to complete the questionnaires; had no severe or disabling pathology; and that they sign the informed consent form. The study had been approved by the Ethics Committee.

All patients were examined individually by a psychologist at 4 different times, as follows: the preoperative stage (within 7 days before surgery) and postoperative (at 1, 6, and 12 months after surgery, coinciding with the patients’ postoperative follow-up examinations).

### 2.4 Statistical analysis

The data were analyzed with the use of the IBM-SPSS 20.0 statistical software package (IBM Corporation, Armonk, NY) for Windows PC. The sociodemographic variables of the 3 groups were compared using Pearson's chi-square (marital status, education level, and employment) and the Welch's U and Games-Howell (age) tests. A mixed analysis of variance (3 × 4 design) was also applied to analyze the influence of 2 independent factors on anxiety and quality of life, namely, type of cosmetic breast surgery (breast augmentation, mastopexy, and breast reduction) and time (preoperative stage and at 1, 6, and 12 months postoperative). For effect size indexes, Cohen's *d* (for continuous variables) and Cohen's *w* (for categorical variables) were computed.

## 3 Results

First, comparisons of sociodemographic data were done for the 3 groups in the study (Table 1). The 3 groups of women were homogeneous with regard to marital status (*P* = 0.077), education level (*P* = 0.509), and employment (*P* = 0.140). However, the women operated on for breast augmentation were younger than those in the mastopexy (*P* < 0.001, medium effect size) and breast reduction (*P* = 0.002, large effect size) groups.

No interactive effects between the type of cosmetic breast surgery and time factors were found for anxiety (state or trait), as seen in Table 2. Of the main effects, only the time factor was relevant (*P* < 0.001), showing very important statistically significant differences (all of them had a medium effect size) between the preoperative stage and the 3 postoperative stages at 1 month (*P* < 0.001), 6 months (*P* < 0.001), and 12 months (*P* < 0.001). The specific scores on anxiety (state and trait) were higher in the preoperative stage and decreased in the postoperative stage, with no statistically significant differences (*P* > 0.99, null effect size) between the 3 postoperative stages (1, 6, and 12 months).

**Table 2:**
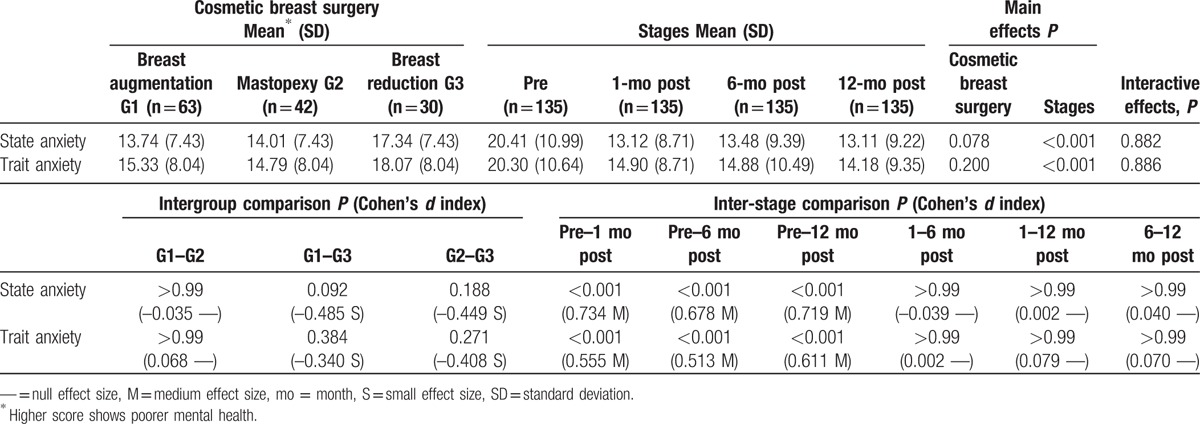
Anxiety symptomatology evolution by type of cosmetic breast surgery.

**Figure 1:**
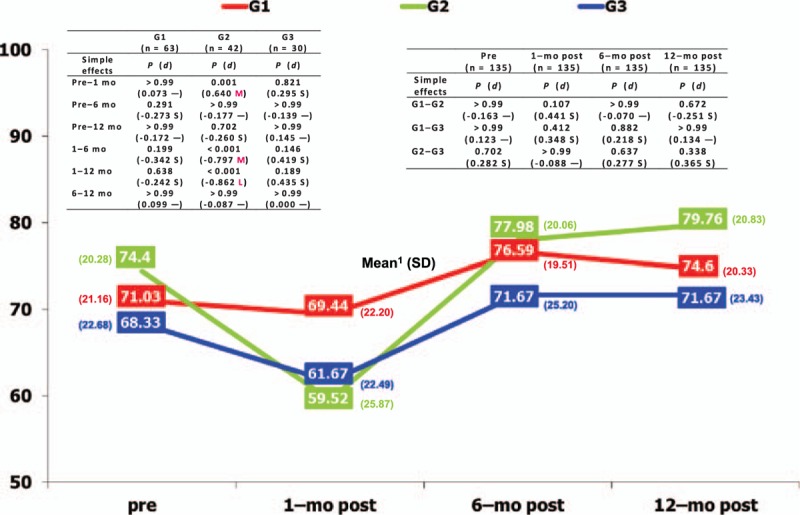
Interactive effects regarding vitality dimension in cosmetic breast surgery. G1 = breast augmentation, G2 = mastopexy, G3 = breast reduction, L = large effect size, M = medium effect size, mo = month, S = small effect size, SD = standard deviation. ^1^Lower score shows poorer quality of life in vitality; — = null effect size.

**Figure 2:**
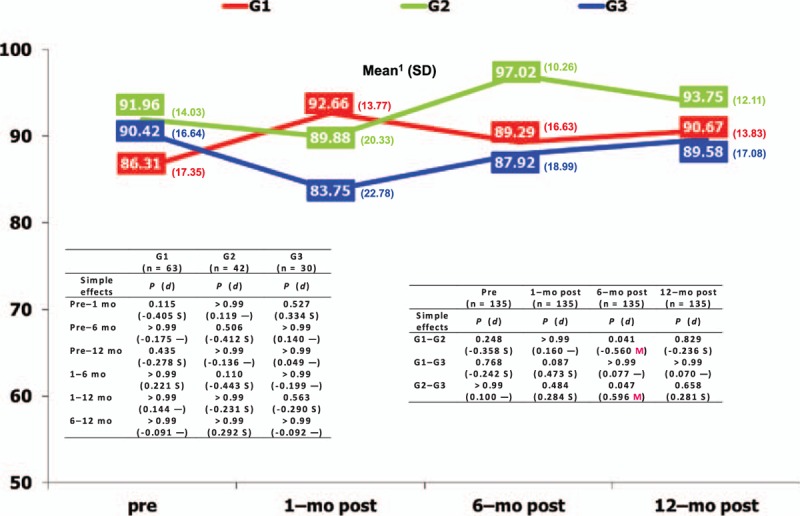
Interactive effects regarding role-emotional dimension in cosmetic breast surgery. G1 = breast augmentation, G2 = mastopexy, G3 = breast reduction, SD = standard deviation, M = medium effect size, mo = month, S = small effect size. ^1^Lower score shows poorer quality of life in role-emotional; — = null effect size.

Interactive effects were found for quality of life between the type of cosmetic breast surgery and time in the vitality (*P* = 0.044) and role-emotional (*P* = 0.023) dimensions, as shown in Table 3. The most relevant simple effects (Fig. 1) showed that time had a significant effect (medium and large effect sizes) on the women's vitality operated on for mastopexy (who felt much worse 1 month after surgery than during the rest of the stages), compared to the other 2 groups. The most relevant simple effects on the role-emotional dimension showed that it was significantly influenced by the type of cosmetic surgery (medium effect size) (Fig. 2) at 6 months postoperative, with women operated on for mastopexy scoring better than those in the other 2 groups.

**Table 3:**
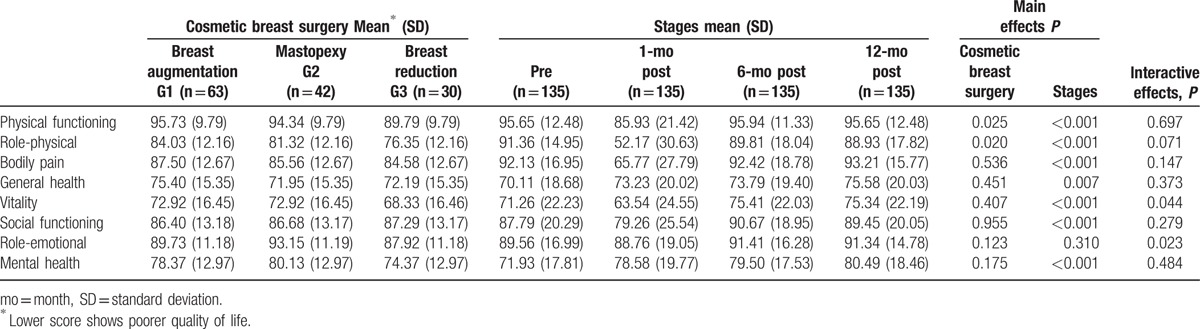
Evolution of quality of life by type of cosmetic breast surgery.

It should be emphasized that for the rest of the quality-of-life dimensions, as shown in Tables 3 and 4, the main effect of type of cosmetic breast surgery was significant in physical functioning (*P* = 0.025) and role-physical (*P* = 0.020). In both, there were very relevant statistically significant differences (medium effect sizes) between the groups of women with breast augmentation and those with breast reduction, who showed worse quality of life. Furthermore, the main effect of time also exerted a significant influence on physical functioning (*P* < 0.001), role-physical (*P* < 0.001), bodily pain (*P* < 0.001), general health (*P* = 0.007), social functioning (*P* < 0.001), and mental health (*P* < 0.001). Focusing specifically on the most relevant effect sizes (medium and large), differences were found in physical functioning, role-physical, and bodily pain at 1 month postoperative, when they felt worse, compared to the rest of the stages, and in the social functioning dimension at 1 month when they also felt worse, compared to 6 months since surgery.

**Table 4:**
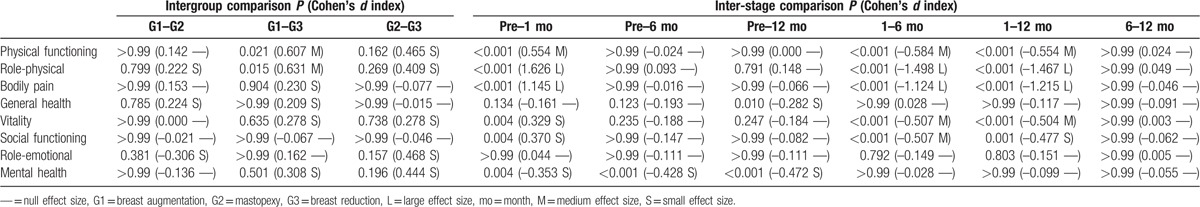
Comparison of quality of life in the 3 groups (breast augmentation, mastopexy, and breast reduction) and 4 stages (preoperative and at 1, 6, and 12 months postoperative).

## 4 Discussion

In this study, we concentrated on analyzing whether time exerted a significant influence on patient anxiety symptomology and quality of life, based on type of cosmetic breast surgery. This proposal is of scientific significance, because although various longitudinal studies have concluded that time exerts a positive influence, there are few studies that analyze the psychological evolution of the patients as a function of the different types of surgery. Therefore, we selected 3 groups of patients (breast augmentation, mastopexy, and breast reduction), among which there were no sociodemographic differences (marital status, education level, and employment) except in the age variable, wherein women who underwent breast augmentation were younger than those in the other 2 groups. This is compatible with findings by other authors, with patients with breast implants proving to be significantly younger than whose who underwent other types of cosmetic breast surgery, including reduction/mastopexy, with mean ages of 42 and 45 years, respectively.^[28]^ Other studies have also found that patients with breast implants underwent breast surgery at a significantly younger age than did breast reduction patients,^[29,30]^ with the typical breast augmentation patient profile being of a young woman aged 28 to 44 years.^[31]^ These differences in age are explained by the fact that most clinical signs for undergoing mastopexy or breast reduction are associated with a series of problems that do not usually appear in younger women, such as sagging breasts due to pregnancy or breast-feeding, hypertrophy after birth or after menopause, and so forth.

When the evolution of anxiety symptomatology (state and trait anxiety) was analyzed by type of surgery, we found that time exerted an identically important influence on all 3 groups, that is, anxiety was higher in the preoperative stage and decreased from the first month after surgery, remaining stable throughout the postoperative period (at 1, 6, and 12 months). These results are in line with other longitudinal studies,^[17,18]^ in which higher levels of anxiety in the preoperative period were associated with fear of the operation itself, anesthesia, postoperative recovery, and even added stress from participants’ desire to improve their body image^[32]^.

It should be stressed that 2 dimensions of quality of life, vitality and role-emotional, evolved differently depending on type of surgery. Compared to the other groups, women who had had a mastopexy showed less vitality after 1 month since surgery than in the rest of the stages (preoperative and at 6–12 months postoperative), and obtained the best scores in the role-emotional dimension 6 months after surgery. These results could be explained by the special characteristics of breast tissue in patients with breast ptosis (mainly their lack of elasticity), the surgical procedure in mastopexy, and especially augmentation mastopexy, compared to other types of cosmetic surgery, with higher postoperative risks, such as nipple loss due to vascular compromise, nipple malposition due to overcorrection, or under-correction of ptosis, infection of implant, visible scars, or loss of sensitivity of the nipple.^[33]^ Therefore, the mastopexy group's vitality may wane in the immediate postoperative period (1 month) due to a higher rate of complications inherent in the surgical procedure, recovering considerably later (postoperative at 6 and 12 months), and favorably influencing the role-emotional dimension more strongly than in the other 2 groups (postoperative at 6 months). This could also explain the poorer quality of life experienced by women who had breast reduction, compared to the breast augmentation group, in the physical functioning and role-physical dimensions. Similarly, the postoperative experience of patients who had had breast reduction was worse than they had expected, and they were unsatisfied either with surgery or with their recovery from it for various reasons, such as more pain than expected, large scars, slow healing, or difficulty sleeping.^[22]^ Among other common postoperative complications after breast reduction are the appearance of hematomas, infection, necrosis of fatty tissue, hypertrophic scars, slow healing, and less sensitivity in the nipple.^[34]^

Breast reduction patients reported being more worried than women with breast augmentation regarding their physical well-being, understood as general physical functioning ability to carry out daily activities both before and after breast surgery.^[35]^

Moreover, it should be mentioned that regardless of type of cosmetic breast surgery, time had the same effect on the physical functioning, role-physical, and bodily pain dimensions. Patients generally felt worse 1 month since surgery, compared to the rest of the stages (preoperative and 6–12 months postoperative). These results are explained by these dimensions being strongly affected by the discomfort and inflammation experienced by the women for several weeks after surgery. This limited their social/family life, and thus also lowered the score on the social functioning dimension during this period (1 month postoperative). However, they recovered their quality of life just as other longitudinal studies on women who previously underwent cosmetic breast surgery have shown.^[2,3,6,8–16,18–20]^

Summarizing, this study showed that cosmetic breast surgery is a therapeutic alternative that, in the long term, when the realistic goals proposed have been reached, is beneficial for personal health, with women recovering their physical and psychological wellbeing. Similarly, another study found that breast reconstruction after mastectomy, like transplant surgery, was very beneficial to patients, with both surgical procedures generating less anxiety-depressive symptomatology and better quality of life than did other operations such as mastectomy.^[36]^ Nonetheless, we think that the following limitations of this study should be taken into account for future research, specifically we suggest some samples for future studies: (a) women who, even when suitable for undergoing any of the 3 types of operations, were not interested in doing so, (b) women who have undergone cosmetic surgery other than breast, and (c) men, who although less frequently, also require this type of surgery.

In view of the results found in this study, we believe that the implementation of psychoeducational programs based on scientific evidence would be very significant, as it would enable professionals to offer adequate preoperative information to women who have already decided to undergo surgery, as well as those who are still undecided. With this information, women could prepare the strategies necessary to face the reality of symptoms, such as pain control and the physical limitations that the immediate postoperative period could pose, even though most of the women experience improved quality of life and emotional well-being in time.
